# Neutrophil degranulation and severely impaired extracellular trap formation at the basis of susceptibility to infections of hemodialysis patients

**DOI:** 10.1186/s12916-022-02564-1

**Published:** 2022-10-26

**Authors:** Salti Talal, Khoury Mona, Awad Karem, Lerenthal Yaniv, Harari-Misgav Reut, Shemesh Ariel, Avraham-Kelbert Moran, Eitam Harel, Salvatore Campisi-Pinto, Abu-Amna Mahmoud, Colodner Raul, Tovbin David, Bar-Sela Gil, Cohen Idan

**Affiliations:** 1 Oncology & Hematology Division, Cancer Center, Emek Medical Center, 21 Yitzhak Rabin Blvd, 1834111 Afula, Israel; 2 Bruce Rappaport Faculty of Medicine, Technion-Israel Institute of Technology, 320002 Haifa, Israel; 3 Nephrology Department, Emek Medical Center, Afula, Israel; 4 Cannasoul Analytics Ltd, Caesarea, Israel; 5 Biomedical Core Facility, Bruce Rappaport Faculty of Medicine Technion-Israel, Haifa, Israel; 6 Emek Medical Center, Clinical Laboratories, Clalit, Afula, Israel; 7 Emek Medical Center, Research Authority, Afula, Israel

**Keywords:** Neutrophils, Hemodialysis, Infections, Neutrophil degranulation, Neutrophil extracellular traps (NETosis)

## Abstract

**Background:**

Chronic kidney disease patients are at increased risk of mortality with cardiovascular diseases and infections as the two leading causes of death for end-stage kidney disease treated with hemodialysis (HD). Mortality from bacterial infections in HD patients is estimated to be 100–1000 times higher than in the healthy population.

**Methods:**

We comprehensively characterized highly pure circulating neutrophils from HD and healthy donors.

**Results:**

Protein levels and transcriptome of HD patients’ neutrophils indicated massive neutrophil degranulation with a dramatic reduction in reactive oxygen species (ROS) production during an oxidative burst and defective oxidative cellular signaling. Moreover, HD neutrophils exhibit severely impaired ability to generate extracellular NET formation (NETosis) in NADPH oxidase-dependent or independent pathways, reflecting their loss of capacity to kill extracellular bacteria. Ectopic hydrogen peroxidase (H_2_O_2_) or recombinant human SOD-1 (rSOD-1) partly restores and improves the extent of HD dysfunctional neutrophil NET formation.

**Conclusions:**

Our report is one of the first singular examples of severe and chronic impairment of NET formation leading to substantial clinical susceptibility to bacteremia that most likely results from the metabolic and environmental milieu typical to HD patients and not by common human genetic deficiencies. In this manner, aberrant gene expression and differential exocytosis of distinct granule populations could reflect the chronic defect in neutrophil functionality and their diminished ability to induce NETosis. Therefore, our findings suggest that targeting NETosis in HD patients may reduce infections, minimize their severity, and decrease the mortality rate from infections in this patient population.

**Supplementary Information:**

The online version contains supplementary material available at 10.1186/s12916-022-02564-1.

## Background

The prevalence of chronic kidney disease (CKD) is growing worldwide [1]. Patients with end-stage renal disease (ESRD) treated with hemodialysis (HD) are at a substantially increased risk of life-threatening infections [2, 3]. The 3-year mortality rate in patients with ESRD is around 25% [4], while concurrently, infections result in significant morbidity and are the second leading cause of death and the number one cause of hospitalization in patients on dialysis [2, 5]. As observed in the last few years, cardiovascular-related hospitalization rates have improved, but infection-related hospitalization rates showed far less progress in the chronic HD population. Death rates from septicemia are 100- to 300-fold higher than in the general population [3–5]. Indeed recently, the American Society of Nephrology (ASN) and the Center for Disease Prevention and Control (CDC) announced an initiative targeting to reduce the complications of the infection among patients receiving HD treatment [2].

Neutrophils play a crucial role in host defense against bacterial, fungal, and viral infections [6]. Impaired neutrophil function in uremia is believed to increase the susceptibility to infections [5, 7–9]. As previously shown, neutrophils separated from HD patients exhibit impaired oxidative production [10–12], decreased in vitro chemotactic response [13], and reduced bactericidal killing capability [12, 14], while the amount of neutrophils is even slightly higher compared with healthy subjects. While the mechanisms responsible for reduced neutrophil functions are not well understood, several interdependent factors contributing to neutrophil dysfunction were proposed, including metabolic changes, uremic toxins [11], malnutrition and inflammation [10], and the dialysis procedure per se [9].

Neutrophil extracellular trap (NETs or NETosis) is a network of extracellular chromatin fibers bound to neutrophils’ granular cytoplasmic proteins [15]. NETosis is characterized by decondensation of intracellular chromatin and disintegration of the nuclear envelope, which allows the mixing of the chromatin with cationic anti-microbial molecules originating from the granules [15]. When the plasma membrane finally permeabilizes, the microbicidal extracellular traps are released. NETs were initially described as antibacterial defense mechanisms in 2004 [16], while original studies demonstrated that NETosis was a form of cell death distinct from apoptosis, potentially utilized as a last resort in neutrophil anti-microbial defense strategies [15, 17]. NETosis is triggered by microbial (bacterial, fungal, and viral), inflammatory cytokines (e.g., IL-8, IFN-ɣ, TNF-α), and endogenous “sterile” triggers (e.g., nitric oxide, platelets, complement, monosodium urate crystals) [18]. Since NET formation is an essential mechanism in host defense against bacterial infection [15, 16, 19, 20], and the increased risk of infection in HD patients was attributed to several risk factors that contribute to neutrophil dysfunction, yet the ability of neutrophils of HD patients to form NETs and the mechanisms governing neutrophil dysfunction is not elucidated. We thus decided to systematically characterize highly pure circulating neutrophils isolated from HD and healthy control (HC) donors to try and uncover the molecular and cellular mechanisms underlying neutrophil dysfunction in HD patients.

## Methods

### Subjects and ethical approval

Forty healthy control (HC) and 46 ESRD patients participated in this study; each patient underwent HD thrice weekly, and each dialysis treatment lasted 4 h (Additional file 1: Table 1). All participants signed informed consent for blood sampling under the approval of the institutional Helsinki committee (0112-19-EMC). Patients with evidence of acute inflammation, infection, or blood transfusion in the last 3 months were excluded. Blood from all donors (e.g., HC and HD) was collected immediately before the ex vivo experiments or taken from HD patients before the dialysis session from the arterial line (using the same method). On average, 3 to 9 ml of blood samples was taken only using EDTA blood tubes.

### Neutrophil isolation

For neutrophil isolation, blood was drawn from gender and age-matched HD patients before the start of a dialysis session and from HC subjects. Human neutrophils were isolated from 3 ml up to 20 ml of whole blood samples (according to the desired cell numbers per application) in EDTA blood sampling tubes. Highly pure neutrophils were isolated using the EasySep™ Direct Human Neutrophil Isolation Kit (Negative selection, Stem cell kit). On average, every 1 ml of blood yields 1.5–1×10^6^ cells (Additional file 1: Fig. S1A and S1B). Cells were maintained in 2% FCS in RPMI media (biological industries) supplemented with 1% penicillin/streptomycin/glutamine. Neutrophil baseline priming or activation status was evaluated by monitoring the rate of extracellular ROS release before or after phorbol myristate acetate (PMA) induction and the release of extracellular superoxide (Additional file 1: Fig. S1C). For this application, 1×10^5^ of highly pure neutrophils were isolated from whole blood samples from HC and HD patients. The cells were seeded in a 96-well black plate with PBS and 20mM of L-012 (Sigma-Aldrich) with or without stimulation with 100nM PMA in a total volume of 200μl at 37 °C. Rates of superoxide production were monitored by ELISA plate readers (SpectraMax Plus 384 Microplate Reader) at 510 nm for up to 30 min.

### Monitoring real-time intracellular ROS

For the real-time detection of intracellular ROS/RNS production in neutrophils, we used ROS-ID total ROS/Superoxide detection kit (ENZ-51010, ENZO Life Sciences). Neutrophils were isolated from blood samples taken from HC subjects and HD patients. Cells (2×10^6^) were seeded on a 16-mm circular coverslip coated with human fibronectin 50μg/ml (Biological Industries) and incubated for 30 min 37°C, 5% CO_2_, to achieve maximal cell adhesion. The ROS/Superoxide detection mix (2 μl Oxidative Stress Detection Reagent (green) for total ROS detection reagent and 2 μl Superoxide Detection Reagent (orange)) was added to 10 ml of 1× wash buffer. From this reaction mix, 200μl was added to each well for 1h in 37°C, 5% CO_2_. The cells were then washed twice with 1× wash buffer and overlaid on a coverslip for imaging with a wide-field fluorescence microscope (Nikon Eclipse) equipped with a standard green (490/525nm) and orange (550/620nm) filter set. Lipopolysaccharides (LPS) (Sigma-Aldrich) or PMA were directly added, and snapshots were taken at indicated times (e.g., 0, 15, 30, 45 min). Alternatively, according to the manufacturer and kit instructions, the reactions were measured for quantitative measurements of superoxides and H_2_O_2_; the samples were measured by fluorescence-activated cell sorting (FACS) (NAVIOS Analyzer, Beckman Coulter Inc).

### NETosis imaging and nuclear morphology

Neutrophils were isolated from blood samples of HC and HD donors. Neutrophils (1×10^6^) were seeded on a 16-mm circular coverslip in 24-well plates and incubated for 2h in 37°C, 5% CO_2_ for adhesion. The cells were then induced with or without PMA or LPS (100ng/ml) or A23187 for 15 and 240 min. Cells were then fixed with paraformaldehyde (PFA, 2% w/v) for 10 min, washed with PBS, and permeabilized with Triton X-100 (0.1% v/v) for 5 min. Staining with Sytox green was performed by incubation for 15–30 min, followed by 2–3 times washing with a phosphate-free buffer (Hanks Balanced Salt Solution Cat. No.14025-092). Imaging was done using a wide-20 field fluorescence microscope (Nikon Eclipse) equipped with a standard green (490/525nm) filter set.

### RNA extraction and sequencing

Total RNA was purified from 6×10^6^ highly pure neutrophil pellets using the RNAEasy Mini kit (QIAGEN), including the DNASEI on-column step for applications (e.g., for qPCR validations or RNA-seq). RNA concentrations were measured using a Qubit 4 Fluorometer (Thermo Fisher Scientific), and RNA quality was measured using Agilent 2100 Bioanalyzer system (Agilent). RNA sequencing libraries were prepared using the CEL-Seq2 protocol, as previously described by Hashimshony [21], with minor modifications. Instead of single cells as input, 2 ng of purified RNA was taken as input for library preparation. The CEL-Seq2 libraries were sequenced on an Illumina NextSeq 550 sequencer (Illumina). RNA measurements, library preparation, and sequencing were performed by the Technion Genome Center, Technion, Israel.

### Transcriptome data analysis

The RNA-Seq raw reads were first quality assessed using FastQC v0.11.9 and subsequently trimmed 10 bp using Trimmomatic [22] v0.39. The trimmed reads were mapped to the human genome (GRCh38.p13) using STAR [23] v2.604a with default parameters and quantification mode of gene counts. Read counts per gene were created using featureCounts v2.0.0 [24] (using the “-t gene” option). Differential expression analysis was performed in R (version 4.1.0) using the package DESeq2 (v1.32.0) [25]. When comparing the HD group to the HC group, genes with a fold change greater than 1.5 (lfc > 0.6) and *P*-value less than 0.01 were defined as upregulated, and genes with a fold change less than 0.65 (lfc < −0.6) and *P*-value less than 0.01 were determined as downregulated. Plots were done using DESeq2 and ggplot2 R libraries. Gene set enrichment analysis was done using ShinyGo (v0.66) [26].

### NETotic cell detection and classification tool

NETotic cell detection and classification were implemented as deep and machine learning models, respectively, in Python. For brevity, instance cell and nucleus segmentation were performed using a pre-trained deep learning model. Then, physical features were extracted for each segmented cell, e.g., perimeter, integrated density, Heywood circularity factor, and area [27]. The segmented cells were manually classified as one of the four categories of lobulated, delobulated, diffused NETs, and spread NETs. A supervised model based on Random Forest Classifier was trained (70% of data) and validated, achieving approx. 90% of accuracy. The trained model was combined with an entire, automated pipeline of cell segmentation and classification for morphology prediction.

### Flow cytometry

Peripheral blood was withdrawn from HC donors and HD patients into EDTA collecting tubes. Purified neutrophil cells were incubated with 5μl of the following conjugated mouse anti-human monoclonal antibodies; CD45-PE, CD11b-FITC, and CD15 (MMA)-FITC were purchased from BD Biosciences, CD18-APC (Biolegend), and CD66b-APC (Invitrogen) for 10 min at RT cells, centrifuged at 1500 RPM for 5 min at RT. Cells were resuspended in PBS before the analysis by NAVIOS flow cytometer Analyzer (Beckman Coulter Inc). The mean fluorescence intensity signals (MFI) were recorded on a minimum of 100,000 cells. The purity of neutrophil isolation by the negative selection cell sorting with the magnetic beads using the EasySep™ Direct Human Neutrophil Isolation Kit (Negative selection, Stem cell kit) was assessed according to the kit instructions using anti-CD66b-APC (Invitrogen), CD16+ (Backman Coulter Inc) (Additional file 1: Fig. S1A).

### Sytox green NETosis quantification

Neutrophils were isolated from whole blood samples of HC and HD patients as described above. Cells were centrifuged at 2000 rpm for 10 min at RT and seeded at 1×10^5^ cells per well in 96-well plates with a final volume of 150 μl. Cell supernatants contain cell-free DNA (cfDNA) before or after adding 100nM PMA, 2.5 mM A23187 (Sigma) for 3h at 37°C in cell media. Indicated units of recombinant human SOD1 Protein (Sino Biological) were added together with PMA. cfDNA was quantified using a rapid fluorometric assay previously described [16]. Briefly, the non-cell-permeant DNA binding dye Sytox green (Molecular Probes) was added at a final concentration of 5μM to detect extracellular DNA to 10μl of cell supernatant containing the NET cfDNA diluted in a total volume of 200μl PBS. DNA standard (plasmid DNA) in a total final concentration ranging between 0.1 and 5ng/ml was used as DNA standard, and non-stimulated neutrophils were used as control. Fluorescence was measured with the 96-well Microtiter Plate Reader at an emission wavelength of 535nm and an excitation wavelength of 485 nm (infinite 200Pro). Each experiment was performed in biological triplicates for HC or HD and presented as means −/+ standard deviation (SD) of relative fluorescence units (RFU).

### Protein extraction and western blotting

Proteins were extracted from 1.5×10^7^ neutrophils suspended in 120μl of lysis buffer (50mM Tris-HCl pH=7.5, 1% SDS, 10% (v/v) protease inhibitors (p8340, Sigma-21 Aldrich, MO), 1% Triton X100, 150mM NaCl) and incubated for 30 min on ice. Protein concentrations were determined for each sample with Bradford assay (BioRad), and equal protein loads were supplemented with 5× Laemmle sample buffer for a final concentration of 1× and boiled for 10 min at 100 °C. Total cell protein extracts were separated over 12 or 15% SDS-PAGE and transferred to a nitrocellulose membrane (Whatman). Membranes were blocked with 5% BSA, incubated with specific antibodies against human PAD4 (ab96758; Abcam), Anti-Neutrophil Elastase (G-2; SC-55549; Santa Cruz Biotechnology, Inc.), Anti-MPO/Myeloperoxidase (266-6K1; SC-52707; Santa Cruz Biotechnology, Inc.), MMP-9 (2C3; SC-21733; Santa Cruz Biotechnology, Inc.), and the housekeeping protein GAPDH (Cell Signaling 2118S) as a loading control. Antibodies were diluted in TBST 1% BSA and incubated for 1 h, followed by incubation with secondary antibody conjugated to horse reddish peroxidase (HRP) (Jackson). Signals were visualized in G-box (Syngene) (Additional file 2).

### Bacterial killing

Human neutrophils were resuspended at 1×10^6^/ml and adhered to plastic plates in neutrophil growth media containing PMA or 0.03% H_2_O_2_ for priming of NETosis. After 30 min incubation at 37 °C, the medium was carefully replaced with fresh medium (to avoid effect on bacterial growth), with (extracellular killing) or without (total killing) cytochalasin D (10 μg/ml) and incubated further for 15 min before Incubation with bacteria. To show that extracellular bacterial killing is derived only by NETosis, samples were pre-incubated with 100 U/ml of RNase and proteinase-free DNase I (Thermo Scientific) to remove extracellular NETs before adding bacteria in a ratio of 1:1 of exponentially grown bacterial culture of *Escherichia coli* (*E. coli*) or *Staphylococcus aureus* (*S. aureus*) in rich bacterial growth media Luria Broth (LB). Samples were then centrifuged at 700*g* for 5 min and incubated at 37 °C and 5% CO_2_ for 1h. The experiments were finalized by serial dilution of each sample on agar plates resulting in a colony-forming unit (CFU) calculation for each triplicate sample. The bacterial killing was then calculated with respect to the number of CFU results from the control sample of bacterial culture incubated alone in media without neutrophils for 1h. The results are presented as the killing percentage of the total live bacteria of the control sample and expressed as means −/+ SD of three independent experiments. The *E. coli* and *S. aureus* bacterial stock cultures were kindly gifted by our institute’s Clalit clinical microbial laboratories at Emek medical center and were purchased from ATCC.

### Quantification and statistical analysis

Routine blood tests from HD patients and HC donors were collected from patients’ electronic medical records (EMR). Measurements were expressed as average and SD of cell count per microliter. Differences between groups (e.g., pre-dialysis VS post-dialysis) were tested by means of paired *t*-test at a *p* < 0.05 significance level. We used the R software program (2020) for statistical analysis.

## Results

### Comparable counts of circulating immune cells between HC and HD donors

**Fig. 1:**
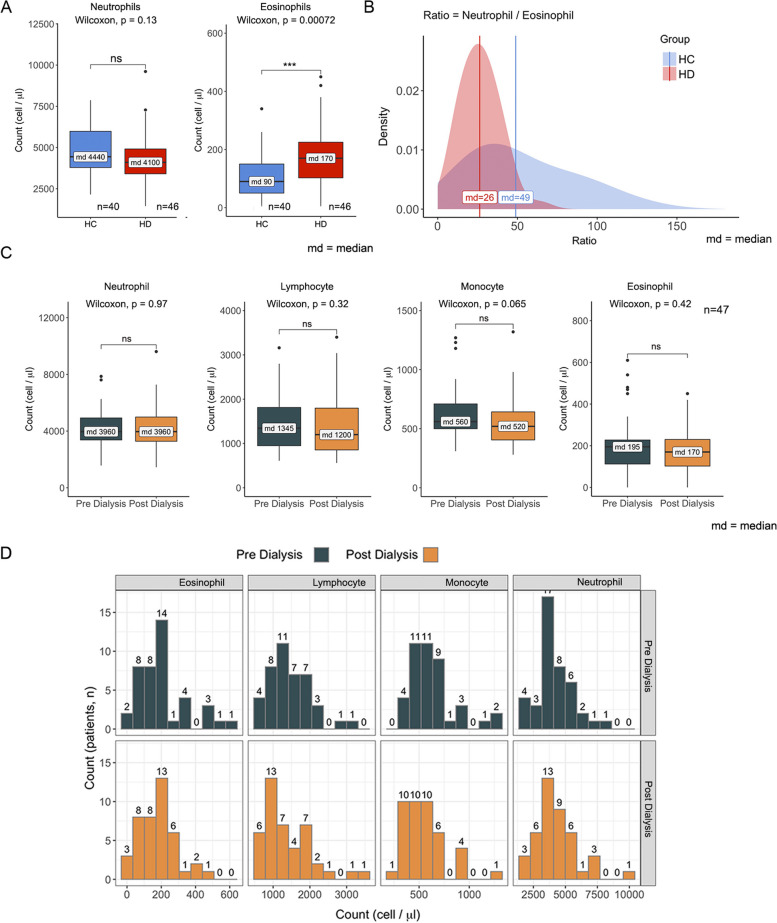
Blood plasma components: in **A** neutrophils and eosinophils, HC patients (*n*=40) versus HD patients (*n*=46), in **B** the distribution of neutrophils/eosinophils ratio (as estimated density) and the corresponding median values; in **C** pre-and post-dialysis count (*n*=47) of neutrophil, lymphocyte, monocytes, and eosinophils, in **D** the histograms

As an initial step to systematically characterize neutrophil functions in HD patients (Additional file 1: Table 1), we first examined the homeostatic levels of neutrophils and eosinophils of HD patients compared to HC. We analyzed routine blood tests taken for 40 HD and 46 HC donors and found no statistical difference between the two groups in the levels of circulating neutrophils (Fig. 1). Surprisingly, we detected an x1.88 increase in HD eosinophils compared to HC, with Neut/Eos’ ratio being two times higher than HC levels (Fig. 1A, B). Next, we tested the possibility that immune cells may be severely damaged during the hemodialysis procedure. We compared blood tests taken from 42 HD patients, pre-and post-dialysis, and compared the average levels of neutrophils, eosinophils, lymphocytes, and monocytes. We observed no significant change in any tested immune cells (changes ranging between 5 and 15%) (Fig. 1C, D). Since no detectable significant differences were observed between the homeostatic levels of any immune cells, we explored the possibility that chronic immune cell dysfunction may be involved in the susceptibility of HD patients to infections.

### Neutrophil degranulation and aberrant transcriptome of HD patient’s neutrophils

We established an *ex vivo* neutrophil cell sorting based on the negative selection of whole blood cells [28]. As previously reported [28], it yielded a highly purified viable non-activated neutrophil cell pellet (Additional file 1: Fig. S1). This superior method was devoid of contaminating eosinophils, allowing the generation of sensitive, more accurate molecular and biochemical data of non-activated cells.

**Fig. 2:**
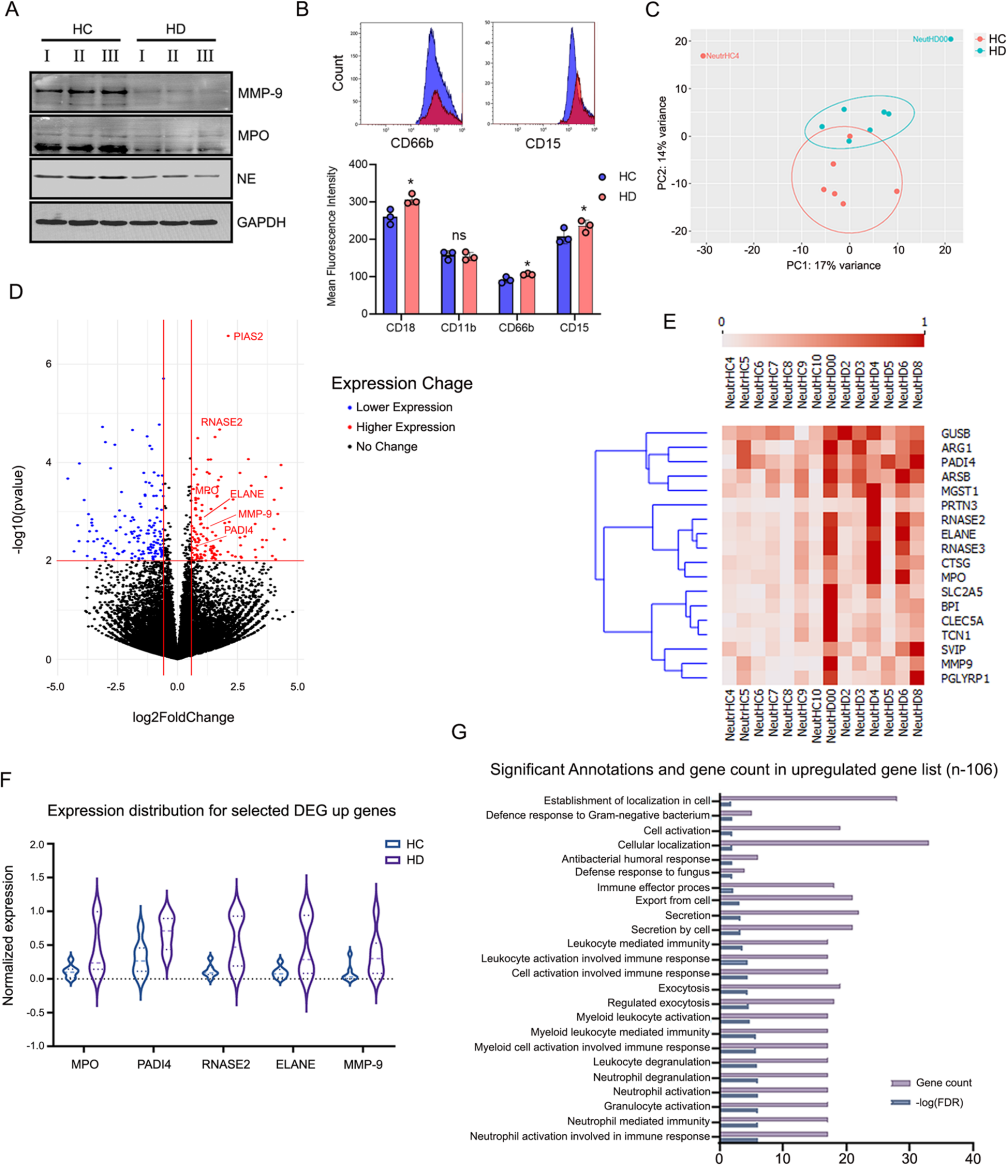
Massive neutrophil degranulation and inadequate gene expression of HD neutrophils. **A** Western blot analysis of steady-state levels of the granules proteins MMP-9, MPO, and NE from highly pure neutrophil pellets taken from HC and HD donors**.** Total proteins were extracted from 3 HC or HD donors; GAPDH was used as a loading control. **B** Elevated levels of neutrophil degranulation surface markers CD18, CD11b, CD66b, and CD15 in HD compared to HC neutrophils. Overlay flow cytometry histogram plots of individual representative HC (blue) and HD (red) neutrophils derived from scatter plots showing levels of CD66b or CD15 (top panel). Mean fluorescence intensities of CD18, CD11b, CD66b, and CD15 in HD compared to HC donors (lower panel, *n*=4). **C** PCA analysis of RNA-Seq expression profile of 7 HD and 7 HC patients. **D** *P*-value and fold change (log2) filters for differential gene expression analysis of HC Vs. HD samples. HD upregulated genes are marked in red, and downregulated genes in blue. **E** Heatmap of selected gene set with neutrophil activation and NETosis-related functional annotation found in HD up-reg. Dataset. Expression levels are normalized between 0 and 1. **F** Normal differential expression distribution on five major NETosis functional genes showing a significantly higher expression in HD samples compared to HC donors. **G** Gene set enrichment analysis (GSEA) annotations and corresponding *p*-values found HD upregulated gene set to be enriched in neutrophil activation and degranulation-related genes

To test if neutrophil cells of HD patients are dysfunctional, we assessed the overall total levels of neutrophil granule proteins such as myeloperoxidase (MPO), neutrophil elastase (NE), and matrix metallopeptidase 9 (MMP-9). These essential anti-microbial proteins are localized mainly to the Azurophil or “primary neutrophil granules” and participate in many anti-microbial defense mechanisms [29–32]. Comparing protein extracts of neutrophils isolated from HC and HD by western blot (WB), we observed a marked depletion of MPO, MMP-9, and NE in HD patient-derived cells (Fig. 2A), as commonly observed after neutrophil degranulation in which neutrophil cytoplasmic granules are mobilized to fuse with the cell membrane resulting in the exocytosis of soluble granule proteins and exposure of membrane at the cell surface. To further corroborate the possibility that HD cells may show changes in neutrophil cell-surface protein composition and due to cytoplasmic granule mobilization [33], we assessed the levels of the primary, secondary, and tertiary granule markers [33]. CD66b, CD15, CD18, and CD11b on neutrophil cell surface taken from HC and HD donors. Apart from comparable levels of CD11b, all other surface markers (e.g., CD66b, CD15, CD18) exhibited significantly higher intensities in HD cells compared to HC neutrophils (Fig. 2B). Since it has been reported that progressive loss of granule content and changes in the neutrophil transcriptome [34] and proteome may reduce the NET-forming capacity [35], we generated a set of differential gene expression profiles, comparing 7 HC and 7 HD patients (Fig. 2C) by RNA-seq to try and uncover novel intracellular signaling, cellular networks, or molecular transcriptional networks compensating for inadequate gene expression in HD neutrophils. Comparing differences in gene expression between HD and HC neutrophils showed a distinct difference between the transcription maps (Fig. 2C and Additional file 1: Fig. S2). Surprisingly, counter to our initial expectations, the distribution of HD under-expressed genes was scattered sporadically with no significant gene type enrichment, while the HD upregulated genes showed a focused classification towards protein-coding genes (Fig. 2D and Additional file 1: Fig. S2A). In-depth analysis of the HD upregulated differentially expressed genes (Additional file 1: Fig. S2B) showed a significantly higher expression of essential neutrophil genes involved in antibacterial defense and NET formation like MPO, NE [31], MMP-9, RNASE2 [36], and Peptidyl Arginine Deiminase 4 (PADI4) [19] (Fig. 2D–F). Interestingly, in line with the higher CD66b levels observed in HD neutrophils, even though below expression cutoff levels, CD66b transcripts were significantly elevated in HD compared with HC cells (Additional file 1: Fig. S2C). Furthermore, functional gene set enrichment analysis on the HD upregulated gene list found significant enrichment of genes in several anti-microbial mechanisms and neutrophil or leukocyte degranulation annotations (Fig. 2G), while no significant functional annotation was identified on the HD downregulated gene set. Together, these results suggest that aberrant gene expression and differential exocytosis of distinct granule populations could reflect the chronic defect in neutrophil functionality and their diminished ability to induce NETosis [33, 35].

### Severely impaired NOX-independent NETosis of neutrophils taken from HD patients

**Fig. 3:**
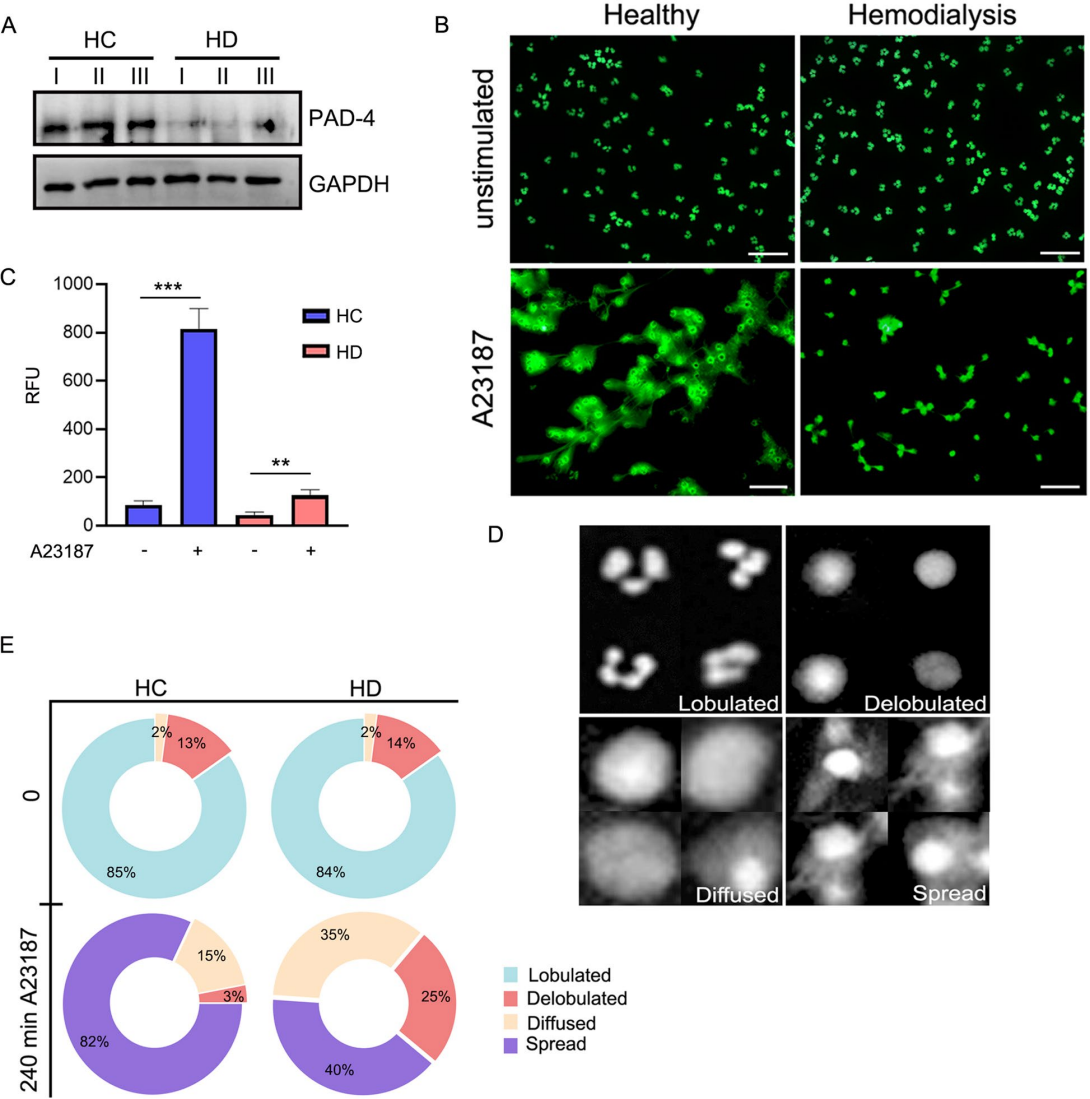
Neutrophils of HD patients show dramatically diminished NOX-independent NETosis with a significant interruption in chromatin decondensation. **A** Western blot analysis of steady-state levels of the PADI-4 enzyme in HC and HD donors neutrophils. **B** NETosis assay and visualization of NET deployment by neutrophils of HC (left) or HD (right) stimulated with Calcium Ionophore A23187 for the stimulation of the NOX-independent pathway visualized by Sytox green in fluorescence microscopy. **C** Quantification of supernatant cfDNA of neutrophils of HC and HD donors stimulated with A23187 for 3 h. The remaining cells were plated, and supernatant cfDNA was quantified by a rapid fluorometric assay using Sytox green. Results are presented as means ± S.E. of RFU *n*=6. **D** The machine learning image analysis tool for quantifying NETosis and chromatin architecture shows sample snapshots of the four neutrophil nuclear stages of NETosis as lobulated, delobulated, diffused, and spread NETs. **E** Overall image analysis of 100 cells per donor out of 6 total donors for each group (e.g., HC or HD). With no stimulation, HC and HD cells show no difference in distribution when 85% of the nuclei are defined as Lobulated. After 3 h stimulation with A23187, 82% of HC cells were defined as spread NETs in HC while only 40% in HD cells. Importantly, in HD cells, a large portion of cells is defined as Delobulated, Diffused as an indication of interruption in chromatin decondensation during NOX-independent NET formation

Since histone hyper-citrullination and chromatin decondensation mediated by PADI4 are essential first steps of the innate antibacterial immunity mediated by NETosis [19], we tested the levels of the PADI-4 enzyme to examine if its overall levels are in correlation with our RNA-seq. Probing protein levels of PADI-4 by WB using total cell proteins of 3 HC and HD donors exhibited a remarkable reduction in PADI-4 steady-state levels of HD cells (Fig. 3A). Surprisingly, again, PADI-4 protein levels in HD neutrophils showed an inverse correlation to its transcript levels. These diminished PADI-4 protein levels in HD cells may dysregulate the first steps of NETosis (chromatin decondensation) and could lead to severely impaired NETosis of the NOX-independent pathway. To test this possibility, we established a microscopic assay for NETosis visualized and quantified using SYTOX green. We developed a fully automatic image analysis tool using a machine learning model to analyze the percentage of NETotic cells under different treatments to assess NET formation using the microscopic assays unbiasedly. Cell annotation was performed based on cell segmentation and labeled images, classifying the cells into four categories: lobulated, delobulated, diffused NETs, and spread NETs, as previously performed in [27]. We induced cells from HC and HD donors with A23187 and visualized NETosis with fluorescence microscopy (Fig. 3B). Uninduced control cells showed no visible differences in nuclear architecture or nuclear morphology when a dramatic reduction in the spread of NETs was viable after 4 h of incubation with A23187 (Fig. 3B). We further confirm these findings by measuring cell-free DNA (cfDNA) released by NETosis with a Sytox green-based assay when marginal elevation in cfDNA after induction could be seen in HD compared to HC cells (Fig. 3C). Next, we performed a measurement of nuclear area and morphology using the four distinct morphologies during the progression to the NET formation (lobulated, delobulated, diffused, and spread NETs) [27] (Fig. 3D) with an automated microscope tool. We analyzed, on average, 100 cells per donor, *n*=6 donors for each group; as per our previous observations, we observed a significant delay in spread NET formation in neutrophils of HD and accumulation of cells in delobulated or diffused nuclear shape (Fig. 3E). In contrast, uninduced cells show no apparent changes in morphology even after 240 min (Additional file 1: Fig. S3A). Collectively, our results show for the first time that neutrophils of HD patients exhibit diminished NOX-independent NETosis with a significant interruption in chromatin decondensation.

### Diminished oxidative burst, defective ROS signaling, and impaired NOX-dependent NETosis by HD patient’s neutrophils

**Fig. 4:**
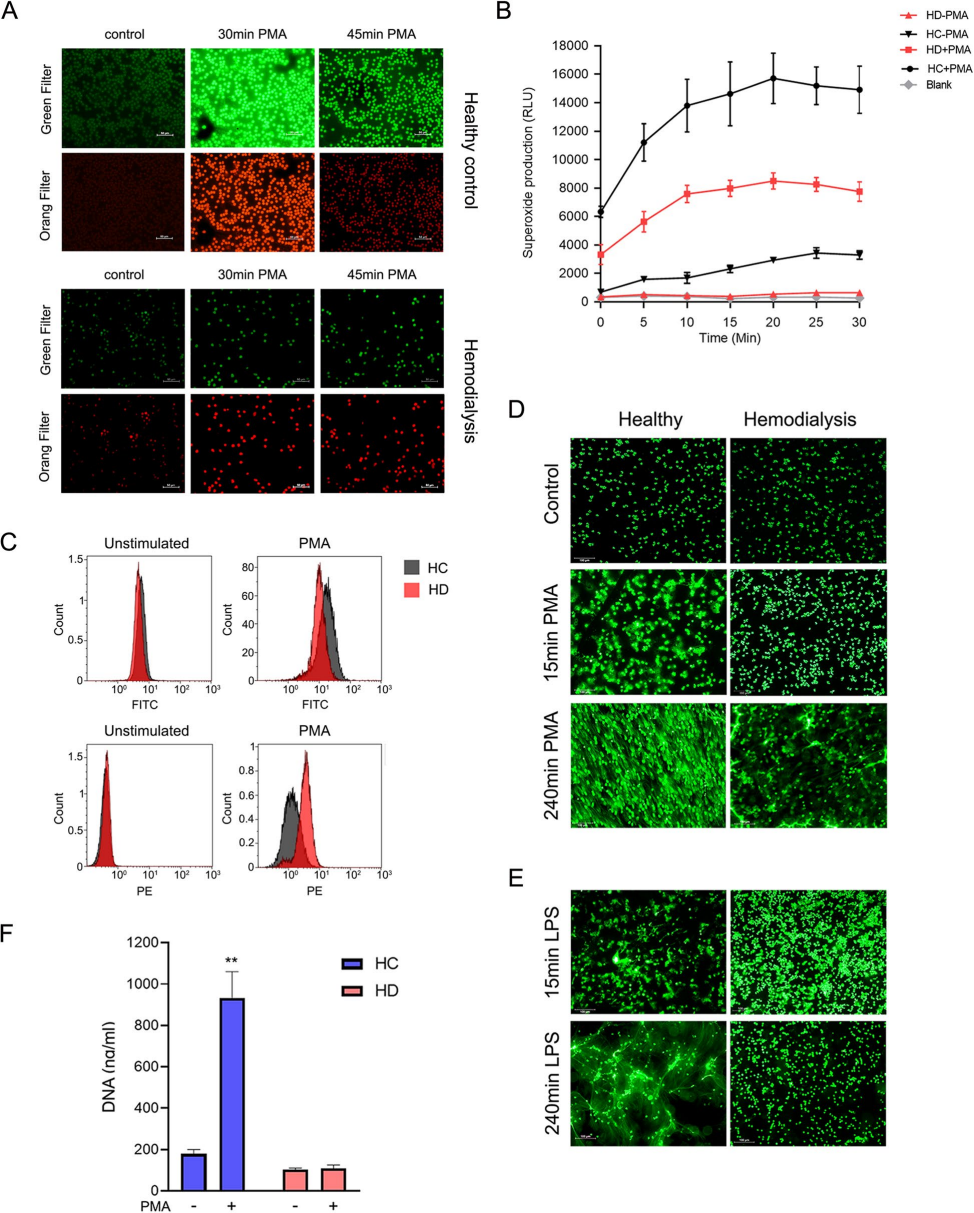
Neutrophils taken from HD patients show malfunctioning ROS signaling and impaired NOX-dependent NETosis. **A** Live-cell monitoring of global levels of ROS/RNS and superoxide in neutrophils induced with PMA. Profiling ROS formation by fluorescence microscopy from HC (top panel) and HD (bottom panel) donors, loaded with ROS/Superoxide detection reagents and activated with PMA. General oxidative stress levels were monitored in the green channel, while superoxide production was detected in the orange/red channel. Snapshots were taken at the indicated time points (e.g., 0, 30, 45 min). **B** Measurement of extracellular ROS release by neutrophils. Neutrophils were isolated from whole blood of HC or HD subjects, supplemented with or without 100nM PMA. Kinetics and rates of extracellular ROS release were measured using L-012 (20 mM) at consecutive times for 30 min using ELISA plate reader chemiluminescence methods at 510nm. Results are presented as means ± S.E. of relative luminescence units (RLU) of six independent donors from each group. **C** Neutrophils of HD patients accumulate superoxides and generate less H_2_O_2_ in the HC cells**.** To quantify live-cell ROS generation, we performed the same experiment as in A but measured the fluorescence of each channel (e.g., FITC**-** H_2_O_2_ or PE-superoxide) using FACS analysis 30 min after PMA stimulation. A representative measurement of HC and HD cells ROS generation is presented out of 3 independent donors from each group. Highly pure neutrophils isolated HC, and HD donors were stimulated with **D** 100nM PMA or **E** 100 ng/mL LPS for 15 min or 240min. Nuclear architecture and NET DNA were stained and visualized with SYTOX Green. Representative images are shown out of 3 independent experiments. **F** Isolated neutrophils were stimulated with or without 100nM PMA for 3 h at 37°C. The remaining cells were plated, and supernatant cfDNA was quantified by a rapid fluorometric assay using Sytox green. Results are presented as means ± S.E. of RFU *n*=6

Neutrophils generate ROS during phagocytosis and respond to soluble agonists. This functional response, termed oxidative burst, contributes to host defense [37]. The white blood cells’ inability to respond effectively to pathogen infections is defined as immune dysfunction or paralysis. Persistent immune paralysis could lead to failure to eradicate primary infections or increase susceptibility to secondary infections. Thus, we tested the rates of ROS generation during oxidative burst compared with HC. Using live-cell monitoring of ROS generation with specific indicators for H_2_O_2_ (Green) or superoxide (Red), we measured the intracellular and extracellular ROS production on HC and HD cells after induction with PMA and the endotoxins LPS (Additional file 1: Fig. S3B and S3C). HD patients’ cells exhibit dramatically diminished ROS generation intracellularly or released to the extracellular space for both stimuli (Fig. 4A, B). Furthermore, FACS analysis on mean intensity levels of H_2_O_2_ (FITS probe) confirmed our microscopic and biochemical analysis and showed a magnitude in the production of the H_2_O_2_ in HC cells compared with HD (Fig. 4C top panel). In contrast, neutrophils of HD patients showed accumulation of superoxide (PE probe) (Fig. 4C bottom panel) as an indication of malfunctioning ROS cascade. While ROS plays a vital role in anti-microbial host defense and inflammation, they are also crucial to the signaling cascades leading to NET release via NADPH oxidase [38, 39]. Moreover, neutrophils of HD patients show a substantial reduction in the levels of NE and MPO, which are both required for PMA, *Candida albicans*, and Group B *Streptococcus* (GBS)-induced NETosis [40]; we then tested the ability of HD cells to produce NETs in the NOX-dependent pathway. As expected, challenging neutrophils of HD patients with stimuli of the NOX-dependent pathway like PMA (Fig. 4D) or LPS (Fig. 4E), we observed a dramatic impairment in the formation of spread NETs. Likewise, measurement of cfDNA in the supernatant of neutrophils induced with PMA showed nearly five times more cfDNA when no significant differences were found in cfDNA of HD cells (Fig. 4F). Our results suggest that differential NOX activity and altered oxidative signaling in these cells may have important implications for oxidant-mediated innate immunity and susceptibility to infections.

### The reduced bacterial killing of HD patient’s neutrophils is linked with impaired NETosis

**Fig. 5:**
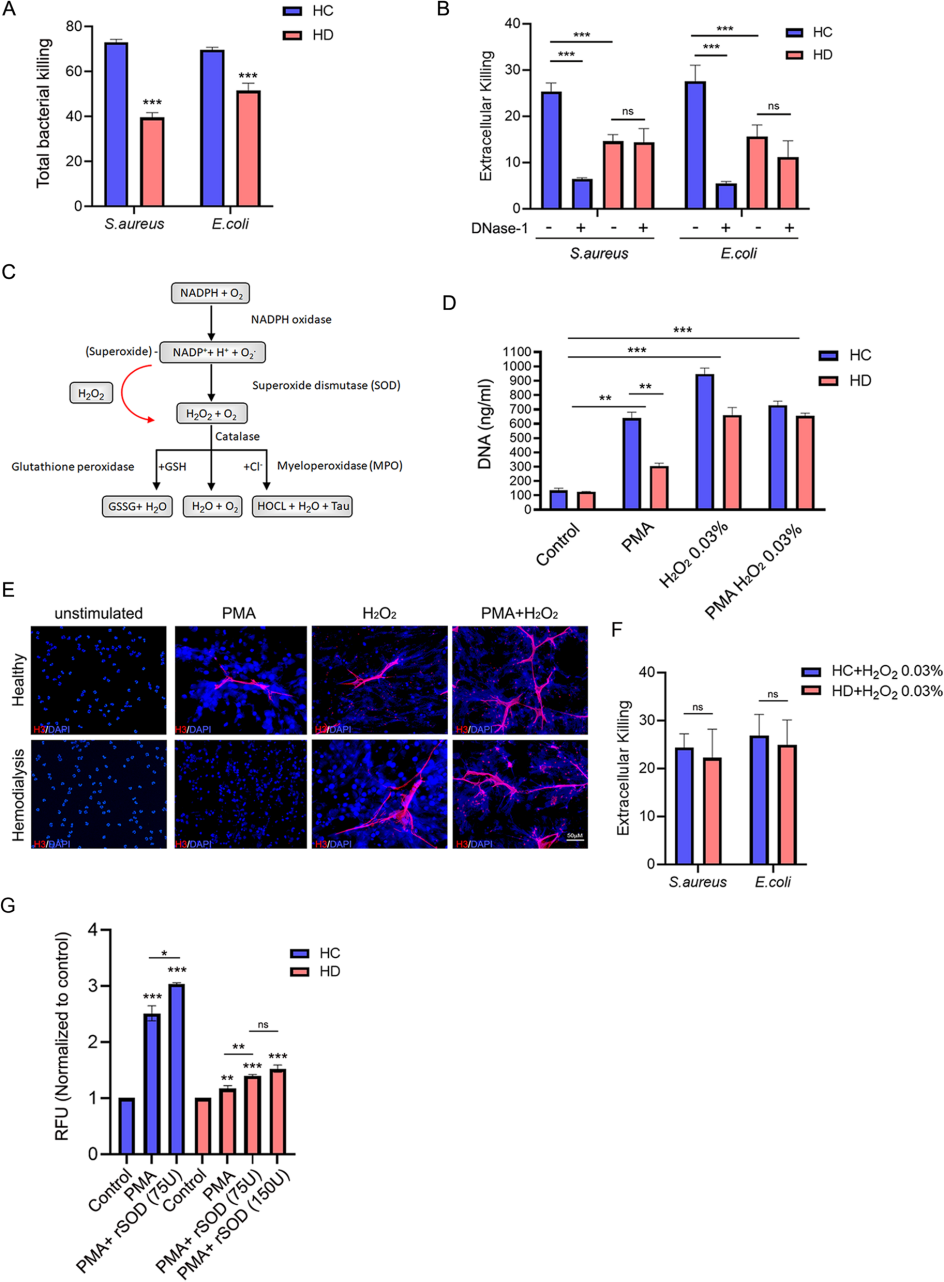
The impaired NET formation of HD neutrophils contributes to diminished bacterial killing and can be restored by H_2_O_2_ or exogenic SOD. **A** Total bacterial killing assay (e.g., phagocytosis and NETosis) with HC and HD neutrophils mixed with either gram-positive (*S. aureus*) or gram-negative bacteria (*E. coli*). Neutrophils and bacteria were mixed in MOI of 1:1 and incubated for 1h. CFU of each bacteria were assessed with serial dilutions, and total killing was calculated compared to a parallel CFU of the control culture (bacteria with no neutrophils). **B** Neutrophils of HC and HD donors were incubated with cytochalasin D to halt phagocytosis. The extracellular bacterial killing (NETosis only) was performed as in **A**. To show that the extracellular bacterial killing derives from NETosis, the same experiment was conducted using RNASE-free DNASE-1. **C** Schematic diagram of ROS signaling during NOX-dependent NET formation. The red arrow indicates the rationality of adding H_2_O_2_ to HD neutrophils and creating ROS signaling bypass. Exogenic H_2_O_2_ restores the NET formation of neutrophils in HD patients. **D** Supernatant cfDNA was quantified by a rapid fluorometric NETosis assay using Sytox green or **E** visualized with fluorescence microscopy. Staining with DAPI was used to visualize nuclei, and extracellular DNA and histone H3 (red) were added to indicate the release of histone associated with the spread NETs. Representative images are shown as merges of the blue and red channels. **F** Pre-incubation H_2_O_2_ restores the bacterial killing capacity of neutrophils taken from HD patients. HD and HC neutrophils’ extracellular bacterial killing capacities were assessed as in B when cells were pre-incubated with Cytochalasin D and then exposed to 0.03% H_2_O_2_ to induce NET formation. The cells media were removed to avoid effects on bacterial viability, and cells were supplemented with a fresh culture medium containing either gram-positive (*S. aureus*) or gram-negative (*E. coli*). CFU of each bacteria were assessed with serial dilutions, and total killing was calculated compared to a parallel CFU of the control culture (*n*=6). **G** Exogenic recombinant SOD-1 improves the NET formation of neutrophils in HD patients. Supernatant cfDNA was quantified by a rapid fluorometric NETosis assay using Sytox green. In all NETosis, fluorometric assay results are presented as means ± S.E. of RFU *n*=4

To understand the impact of degranulation and impaired NETosis on the anti-microbial activity of HD patient’s neutrophils, we performed a bacterial killing assay that measured the total killing (phagocytosis and NETosis) with gram-positive (*S. aureus*) or negative (*E. coli*) (Fig. 5A). In agreement with previous reports [14], we detected a near 50% difference in the killing capacity between HC and HD cells (HC 72.93% vs. HD 39.48%) for *S. aureus* and more than 25% difference in the killing capacity of *E. coli* (HC 69.55% Vs. HD 51.43%). As previously demonstrated, Cytochalasin D treatment did not affect NETosis but effectively blocked phagocytosis [16]. We thus performed the same bacterial killing assay in the presence of Cytochalasin D to measure the extent of extracellular killing (Fig. 5B). Rates of extracellular killing by HC cells were consistent with previous NET killing reports ranging from 25.6 to 28.3% for both bacteria. HD neutrophils showed a substantial reduction in the extracellular killing of 40% compared with HC cells (*E. coli* 15% and *S. aureus* 16%). Moreover, performing the same assays in the presence of DNASE-1 abolished HD cells’ ability to kill extracellular bacteria while having almost no effect on HD cells, suggesting almost no NETs were involved (Fig. 5B).

Finally, we tested the possibility that the impaired NETosis of neutrophils of HD patients can be restored or improved by complementing it with exogenic chemicals or biologics such as recombinant proteins. Since we detected an oxidative malfunction signaling within HD cells with the accumulation of superoxide during PMA induction while hypochlorous acid and H_2_O_2_ are known regulators of NETosis in humans [38, 41], we hypothesize that bypassing the jammed ROS signaling using H_2_O_2_ could restore NET deployment if no further disturbance occurs in chromatin decondensation (Fig. 5C). Incubation of neutrophils with little as 0.03% H_2_O_2_ with or without PMA restored NET deployment by HD cells measured both with Sytox green assay or fluorescence microscopy (Fig. 5D, E). Additionally, pre-incubation of neutrophils with 0.03% H_2_O_2_ eradicated the observed differences in bacterial killing capacities between HD and HC neutrophils (Fig. 5F), further supporting the experimental ability of H_2_O_2_ to induce and restore functional NET formation by HD cells. These results suggest that the impaired NET formation by HD cells could at least to some extent be improved or even restored and most probably derived by the defective oxidative signaling in this pathway. We then tested a more visible biological approach that may enhance HD NET formation by adding exogenic rSOD-1. Consistent with previous results with HC neutrophils [41], exogenic rSOD-1 enhanced PMA-induced NETosis in HC cells and gradually also in HD neutrophils (Fig. 5G). Collectively, our results suggest that restoring NETosis is one of the prime physiological processes that should be targeted as a preventative measure to improve and minimize infection in HD patients.

## Discussion

In this study, we took advantage of negative selection cell sorting to systematically compare highly pure human neutrophils taken from HC and HD donors. This method, indifferent to classical human neutrophil isolation protocols, did not result in significant numbers (~5–20%) of contaminating leukocytes (mostly eosinophils) (Additional file 1: Fig. S1). Such contaminating leukocytes, commonly termed “polymorphonuclear leukocytes” (PMNL), are most likely to lead to inadvertently different phenotypes, baseline activation status, and inaccurate or inconsistent results [28, 42, 43]. In our hands, the negative cell sorting method shows greater yield, no baseline activation, and, most importantly, superior cell sorting purity [28] (Additional file 1: Fig. S1). Taking into consideration that eosinophils release significantly greater ROS [44, 45] and may contribute to an inadequate transcriptome, generate NET like structures termed (ETOsis) [46], possess anti-bactericidal activity, and can activate pro-inflammatory responses [47], using the negative cell sorting, we were able to test effectively assess ROS signaling, transcriptome profiles, protein levels, NET formation, and bacterial killing with minimal modifications, in difference to previous studies.

Overall, in our immune cell survey of circulating blood immune cells of HD patients, we found no significant evidence for damage or immune cell erosion over time compared with HC donors (Fig. 1). These results strengthen the notion that immune cell dysfunction governs the diminished ability of HD neutrophils to kill bacteria and may lead to the clinical outcome seen in HD rather than a considerable temporary or prolonged reduction of immune cell counts. The substantial neutrophil degranulation and intracellular reduction of key antibacterial proteins such as MPO, NE, and MMP-9 may result from persistent immune activation by uremia, the HD procedure, or the low-grade chronic inflammation seen in HD patients. Indeed, serum NE [48] and MPO [49] levels are highly elevated and correlate with levels of markers of inflammation and prospective mortality risk in HD patients. Moreover, MPO levels are significantly higher after HD than pre-dialysis levels, suggesting HD treatment “per se” can aggravate immune cell activation and degranulation [50]. Such persistent and chronic immune activation can also explain the discrepancy between the transcriptome and protein levels of the distinct essential antibacterial genes. Although it is decidedly common that neutrophil exhibits dynamic transcriptome-proteome correlation when RNA and proteins show highly differential kinetics and do not align [51], our transcriptome mapping of HC vs. HD cells demonstrated the exact opposite mirror correlation between RNA and protein levels of PADI-4 MPO, NE, and MMP-9 (Fig. 2). These results support the possibility that HD neutrophils experience constant potent immune activation, which leads to substantial degranulation and may try to compensate it with elevated transcript levels of the same vital genes, adapting their transcriptome to the specific biologic status [52, 53]. Nonetheless, HD cells seem to retain some bacterial killing capacity resistant to the inhibition by Cytochalasin D (phagocytosis) or DNASE-1 (NETosis) (Fig. 5B). Therefore, we speculate that HD cells preserve limited degranulation capacity, most probably through remnants of residual levels of NE that enable such killing. This is further supported by the WB levels of NE (Fig. 2A), in which indifference to MMP-9 and MPO is not entirely depleted in HD cells. In addition, although currently, we have no direct explanation for the higher eosinophil counts seen in HD patients, this may also indicate substantial immune activation during the HD process. Interestingly, changes (both low or high) in eosinophil counts were previously associated with higher all-cause mortality in incident HD patients [54].

Neutrophil functional capacity to undergo NETosis can differ with different physiological states, suggesting that neutrophil practical assortment may be clinically relevant to infection susceptibility or result in the inability to clear existing infections. Indeed, several metabolic conditions associated with chronic inflammation were shown to increase neutrophil predisposition to form NETs. For example, neutrophils of diabetic patients [55], systemic lupus erythematosus (SLE) [56, 57], radiographic axial spondyloarthritis (r-axSpA) [58], COPD [59], Kawasaki disease (KD) [60], cystic fibrosis (CF) [61] and rheumatoid arthritis (RA) [62], all displays enhance NET formation [63]. Recently even in different malignancies, enhanced NETosis and NET are mainly characterized and recognized as new factor in tumor progression [64]. On the other hand, impaired NET formation is mostly reported only in a handful of human genetic mutations like chronic granulomatous disease (CGD), an inherited disorder of NADPH oxidase characterized by recurrent life-threatening bacterial and fungal infections when neutrophils from CGD patients are defective in NETosis [65]. On the same line, neutrophils from donors completely deficient in MPO fail to form NETs, indicating that MPO is required for the process [66]. Likewise, a novel ELANE mutation associated with inflammatory arthritis, defective NETosis, and recurrent parvovirus infection was recently described [67]. Reduced NETosis was also reported during systemic inflammation in mice models of Menkes and Wilson disease [68]. Thus, collectively, it seems that massive defects in NET formation manifested with clear clinical implications occur only due to genetic disorders, while reduced NETosis as a result of metabolic or physiological conditions was only reported in temporal acute ethanol exposure [69], patients with liver cirrhosis, or aged individuals [70], all with no clear link to clinical infection. Our results imply that a manifestation of neutrophil degranulation, aberrant gene expression of key neutrophil genes like MPO, NE, and PADI4, and impaired NET formation could severely affect HD patient’s neutrophil bacterial killing capacity and host immunity (Fig. 5).

The sheer impact of NETosis on the host’s immunity and clearance of pathogenic infection in vivo is yet highly debatable. For example, in MPO-deficient mice, the absence of NET formation is associated with fungal dissemination, while this phenotype is not seen with yeast-locked mutants that can be killed by phagocytosis. Another support that NETs block microbial dissemination is based on the finding that bacterial strains with mutations in a NET-degrading nuclease cannot disseminate [71, 72]. On the same line, It is important to note that pathogens like *S. aureus* evolved different mechanisms against host defenses and can degrade NETs to promote evasion [73]. Likewise, *S. aureus* bacterial killing by neutrophils is mediated by a rather specialized rapid NETosis mechanism now defined as vital NET formation [74]. Interestingly, *S. aureus* is recognized as the most common infection and the leading cause of infectious morbidity and mortality among HD patients [5, 75, 76]. Remarkably, aligned with the clinical observations and the fact that *S. aureus* is particularly cleared by NETosis (Fig. 5B), we observed the most significant drop of bacterial killing by HD patient’s neutrophils with the impaired NETosis towards *S. aureus*. In fact, the overall total killing of HD patient’s neutrophils dropped by 50% compared to HC when the parallel killing of *E.coli* only declined by 25% (Fig. 5A). Based on these observations, we believe that restoring NETosis of HD patients is an imminent need with high clinical importance that, at least in part, could promote the reduction of infection incidences in this highly susceptible immunocompromised patient population.

## Conclusions

Our data unveil novel observations underlying the chronic dysregulation of the immune responses by circulating neutrophils leading to HD patients’ immune dysfunction. We demonstrate a massive degranulation, systemic impairment of NET formation, and diminished bacterial killing by HD neutrophils as a cause of chronic uremic milieu, strongly associated with the clinical susceptibility to bacterial infections and mortality. Although the factors contributing to the risk of infection in HD patients are most certainly multifactorial, NETosis impairment is most likely to be one of the primary reasons for recurrent infections among this patient population. Consequently, targeting the enhancement or restoration of NETosis in HD patients may improve clinical outcomes by reducing morbidity and mortality in this high-risk population. Lastly, our data demonstrates one of the first singular examples of chronic and systemic impairment of NETosis linked with clinical susceptibility to infections, most probably resulting from the chronic toxic metabolic environmental causes or periodic immune activation due to the HD procedure and not by genetic deficiencies.

### Supplementary Information

**Additional file 1: Fig S1.** Comparison of neutrophil isolation methods shows the superiority and advantage of negative selection cell sorting. (A) Flow cytometry analysis of cell pellets population separated using dextran, Max exp, or Stem Cell kits. The left column show neutrophil content of lysed whole blood start sample. The Center column shows the final isolated cell pellets of CD16^+^ cells, and the right column shows an analysis of isolated cell pellets with CD16 and CD66b. The CD16^high^ CD66b^high^ cell population is defined as neutrophils, and CD16^low^ CD66b^high^ as eosinophils. (B) Yields and kits efficiency of isolated neutrophil cells per ml blood. (C) Representative measurements of neutrophils' baseline activation result from the isolation/sorting technique. The extent of extracellular ROS release assessed baseline activation before and after stimulation with PMA. **Fig. S2.** (A) Distributions of gene type annotations of differentially expressed genes in HD Upregulated genes (DEG_UP) and HD downregulated genes (DEG_Down). (B) Heatmap of significant differentially expressed HD upregulated genes on all 14 HC and HD samples. The expression level is normalized from 0 (no expression) to 1 (highest expression). **Fig. S3.** No difference in uninduced neutrophil cells of HC and HD patients over 4h. Image analysis of uninduced (with no stimulation) neutrophil cells n=6 donors from each group (e.g., HC or HD). HC and HD cells showed no difference in the distribution of nuclei architecture or any signs of spontaneous NETosis after Incubation of 4h. Neutrophils taken from HD patients and stimulated with LPS generated less intracellular free radicals than HC donors. (B) live-cell measurement of global ROS levels and superoxide in neutrophils induced with 100ng/ml LPS. Profiling ROS formation by fluorescence microscopy was performed with highly pure neutrophils from HC and HD patients, loaded with ROS/Superoxide detection reagents, and activated with LPS. General oxidative stress levels were monitored in the green channel, while the orange/Red channel detected superoxide production. Snapshots were taken in indicated time points (e.g., 0, 15, 45 min). (C) Neutrophils were isolated from whole blood samples of HC and HD. The kinetics of extracellular ROS release was performed with L-012 (20mM) with or without PMA (100nM) stimulation. The kinetics of extracellular ROS release was measured for 30 min using an ELISA plate reader. Results are presented as means ± S.E. of RLU of six independent donors from each group. Neutrophils induced with PMA were a positive control for extracellular ROS release. **Supplementary Table 1.** Hemodialysis patients characteristics.

**Additional file 2.**

## Data Availability

Data will be available upon reasonable request from the corresponding author.

## Abbreviations

ASN: American Society of Nephrology
CDC: Center for Disease Prevention and Control
CF: Cystic fibrosis
cfDNA: Cell-free DNA
CFU: Colony-forming unit
CGD: Chronic granulomatous disease
CKD: Chronic kidney disease
*E. coli*: *Escherichia coli*
EMR: Electronic medical records
ESRD: End-stage renal disease
GBS: Group B *Streptococcus*
GSEA: Gene set enrichment analysis
H_2_O_2_: Hydrogen peroxidase
HC: Healthy control
HD: Hemodialysis
HRP: Horse reddish peroxidase
KD: Kawasaki disease
LB: Luria Broth
LPS: Lipopolysaccharides
MFI: Mean fluorescence intensity signals
MMP-9: Matrix metallopeptidase 9
MPO: Myeloperoxidase
NE: Neutrophil elastase
NETs or NETosis: Neutrophil extracellular trap
PADI4: Peptidyl arginine deiminase 4
PFA: Paraformaldehyde
PMA: Phorbol myristate acetate
PMNL: Polymorphonuclear leukocytes
r-axSpA: Radiographic axial spondyloarthritis
RA: Rheumatoid arthritis
RFU: Relative fluorescence units
RLU: Relative luminescence units
ROS: Reactive oxygen species
rSOD-1: Recombinant SOD-1
*S. aureus*: *Staphylococcus aureus*
SD: Standard deviation
SLE: Systemic lupus erythematosus
WB: Western blot
