# Supplementary material for: Neutrophil degranulation and severely impaired extracellular trap formation at the basis of susceptibility to infections of hemodialysis patients
Source: BMC Med. 2022 Oct 26;20:364. doi: 10.1186/s12916-022-02564-1 (PMC9597999; doi:10.1186/s12916-022-02564-1)
Supplement: Supplementary file 1 — Additional file 1: Fig S1. Comparison of neutrophil isolation methods shows the superiority and advantage of negative selection cell sorting. (A) Flow cytometry analysis of cell pellets population separated using dextran, Max exp, or Stem Cell kits. The left column show neutrophil content of lysed whole blood start sample. The Center column shows the final isolated cell pellets of CD16+ cells, and the right column shows an analysis of isolated cell pellets with CD16 and CD66b. The CD16high CD66bhigh cell population is defined as neutrophils, and CD16low CD66bhigh as eosinophils. (B) Yields and kits efficiency of isolated neutrophil cells per ml blood. (C) Representative measurements of neutrophils' baseline activation result from the isolation/sorting technique. The extent of extracellular ROS release assessed baseline activation before and after stimulation with PMA. Fig. S2. (A) Distributions of gene type annotations of differentially expressed genes in HD Upregulated genes (DEG_UP) and HD downregulated genes (DEG_Down). (B) Heatmap of significant differentially expressed HD upregulated genes on all 14 HC and HD samples. The expression level is normalized from 0 (no expression) to 1 (highest expression). Fig. S3. No difference in uninduced neutrophil cells of HC and HD patients over 4h. Image analysis of uninduced (with no stimulation) neutrophil cells n=6 donors from each group (e.g., HC or HD). HC and HD cells showed no difference in the distribution of nuclei architecture or any signs of spontaneous NETosis after Incubation of 4h. Neutrophils taken from HD patients and stimulated with LPS generated less intracellular free radicals than HC donors. (B) live-cell measurement of global ROS levels and superoxide in neutrophils induced with 100ng/ml LPS. Profiling ROS formation by fluorescence microscopy was performed with highly pure neutrophils from HC and HD patients, loaded with ROS/Superoxide detection reagents, and activated with LPS. General oxidative stress l [file 12916_2022_2564_MOESM1_ESM.pdf]

Salti et al supplementary Fig.1

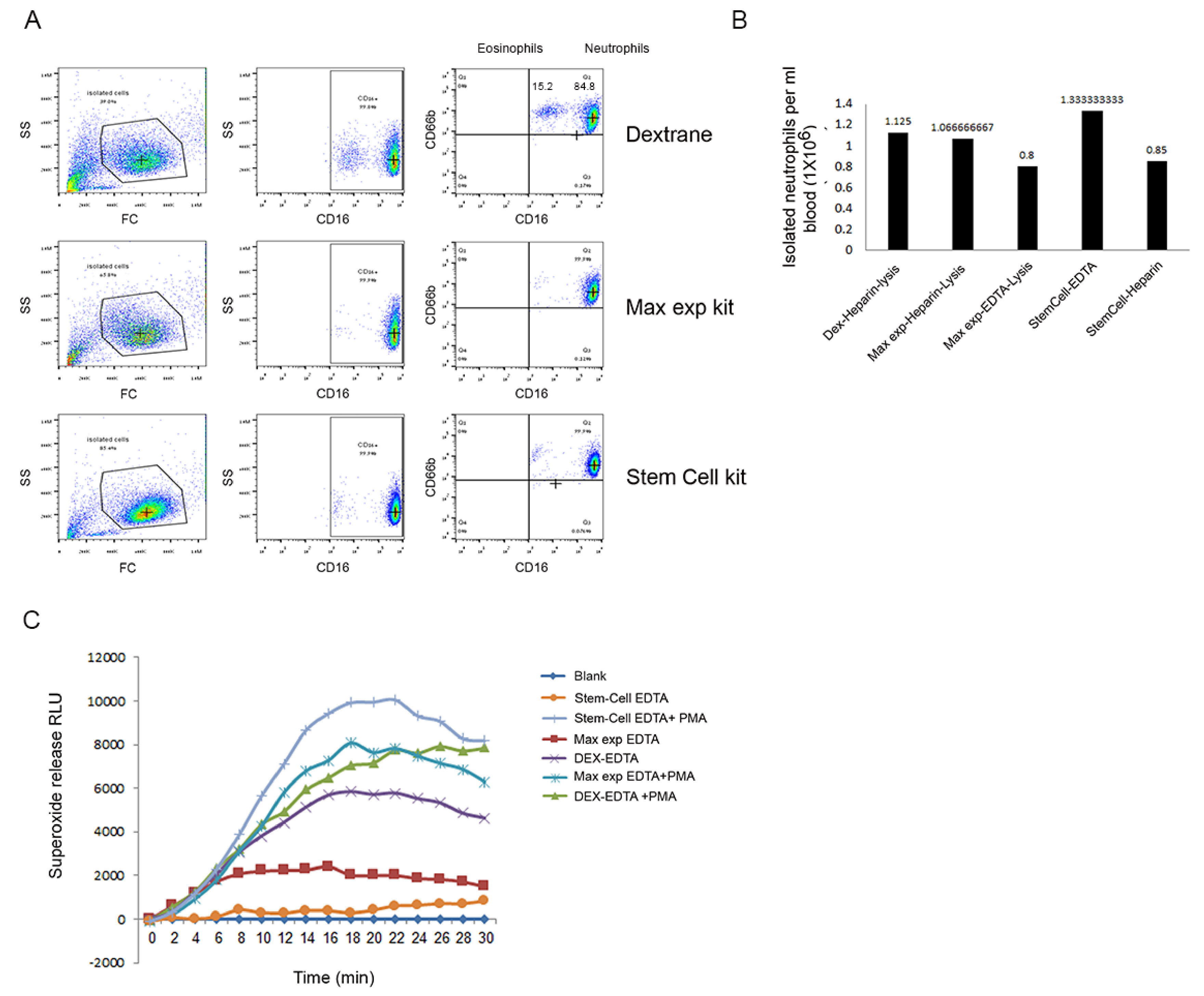

Salti et al supplementary Fig.2

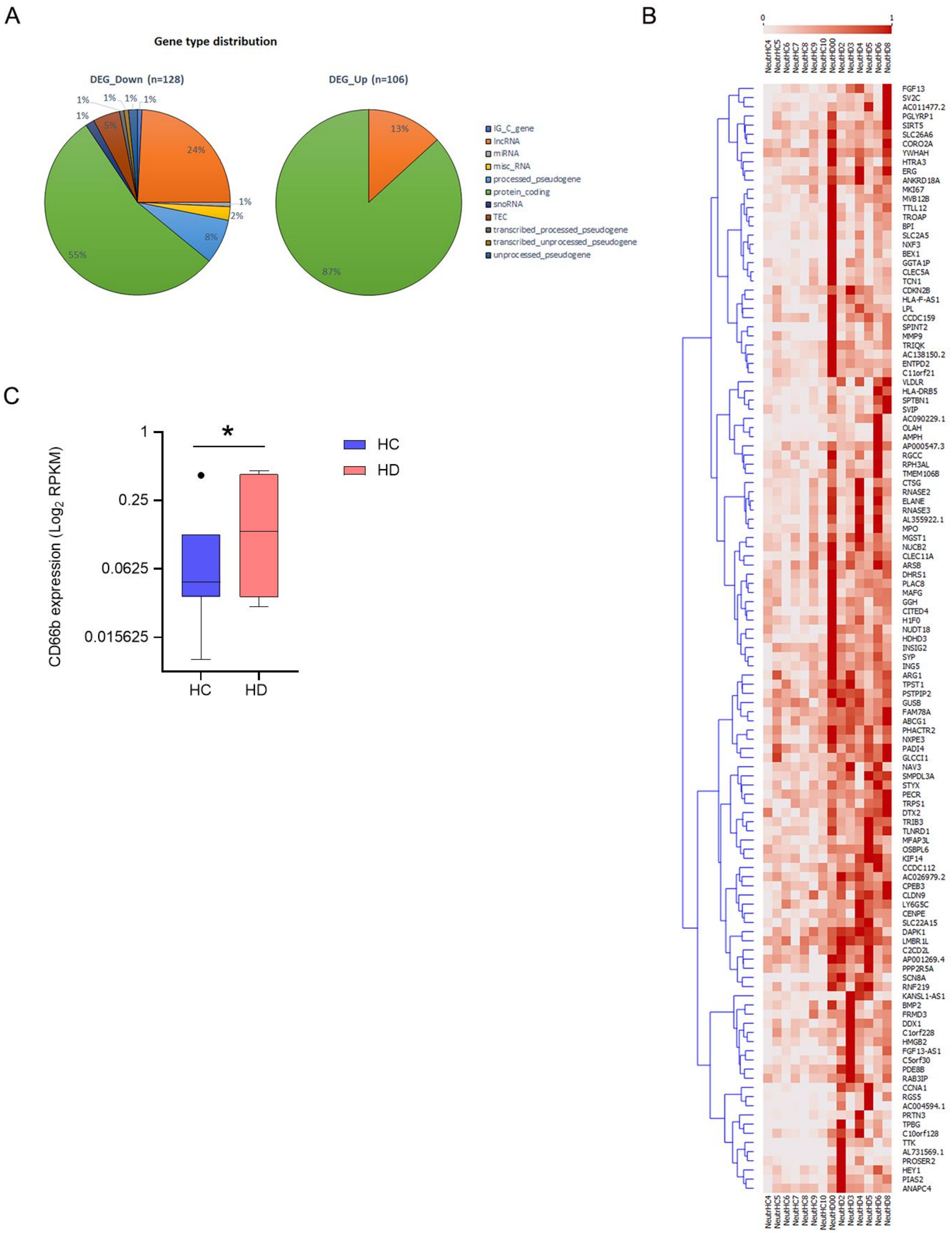

Salti et al supplementary Fig.3

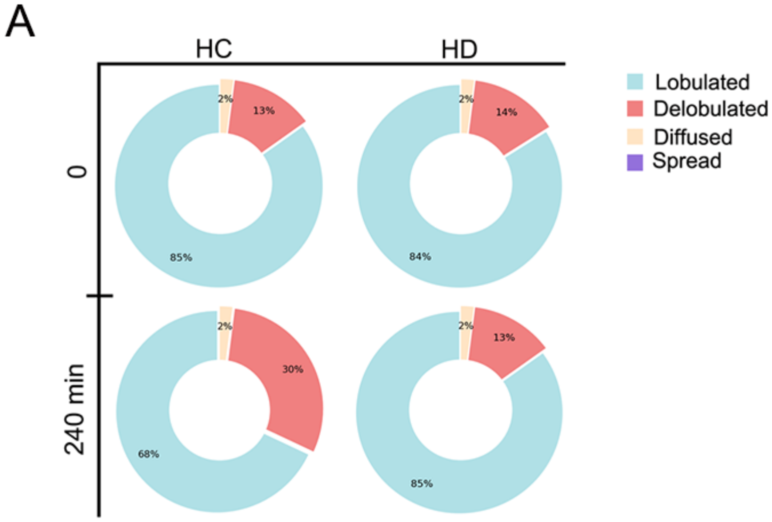

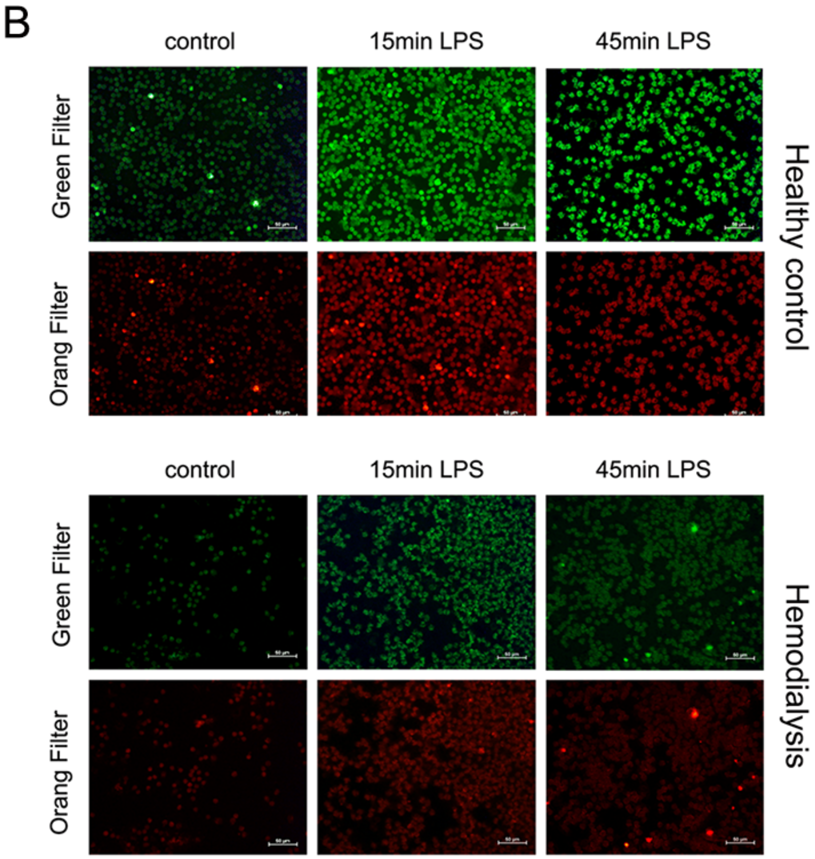

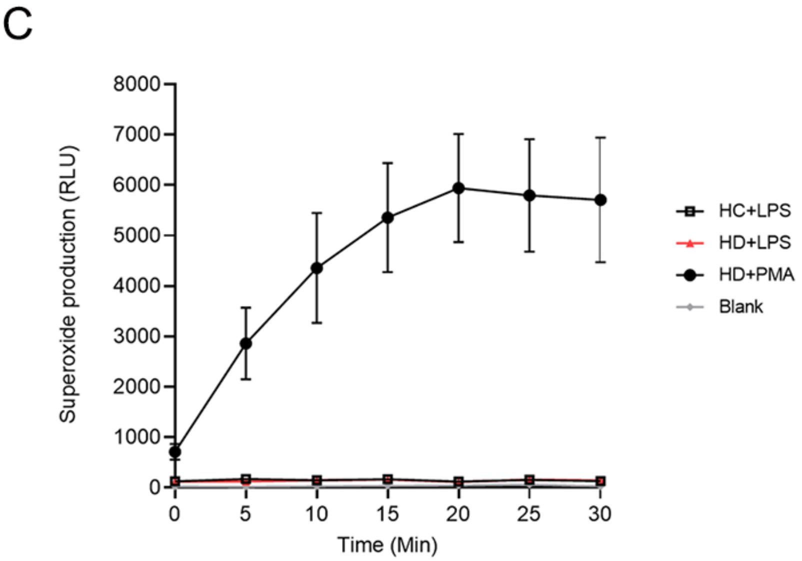

Supplementary Table-1: Hemodialysis patients characteristics

	**Female**	**Male**	**p-value**
**n**	**14**	**32**	
Diabetes = yes (%)	8 (57.1)	21 (65.6)	0.829
Hypertension = yes (%)	11 (78.6)	27 (84.4)	0.956
Lung condition = yes (%)	1 (7.1)	4 (12.5)	0.982
ALBUMIN (mean (SD))	3.64 (0.34)	3.59 (0.32)	0.635
C-REACTIVE PROTEIN (CRP) (mean (SD))	1.90 (2.18)	1.28 (1.98)	0.348
CALCIUM- BLOOD (mean (SD))	8.61 (1.15)	8.71 (0.52)	0.666
CREATININE- BLOOD (mean (SD))	5.99 (1.89)	6.01 (2.21)	0.98
HGB (mean (SD))	11.25 (1.10)	11.13 (1.29)	0.771
LYM% (mean (SD))	19.85 (5.74)	23.37 (13.01)	0.339
LYMP.abs (mean (SD))	1.29 (0.46)	1.84 (2.80)	0.475
NEUT.abs (mean (SD))	4.46 (1.58)	4.14 (1.59)	0.526
NEUT% (mean (SD))	69.25 (8.21)	64.12 (12.64)	0.172
PHOSPHORUS- BLOOD (mean (SD))	5.52 (1.49)	4.99 (1.58)	0.292
PLT (mean (SD))	202.14 (58.74)	192.69 (69.23)	0.658
UREA- BLOOD (mean (SD))	116.07 (38.30)	107.81 (43.57)	0.543
URIC ACID- BLOOD (mean (SD))	6.10 (1.21)	5.64 (1.40)	0.298
WBC (mean (SD))	6.37 (1.94)	6.75 (3.26)	0.691
Years of dialysis (mean (SD))	3.86 (3.61)	2.87 (2.12)	0.25
