# Supplementary figures and images for: Neutrophil degranulation and severely impaired extracellular trap formation at the basis of susceptibility to infections of hemodialysis patients
Source: BMC Med. 2022 Oct 26;20:364. doi: 10.1186/s12916-022-02564-1 (PMC9597999; doi:10.1186/s12916-022-02564-1)

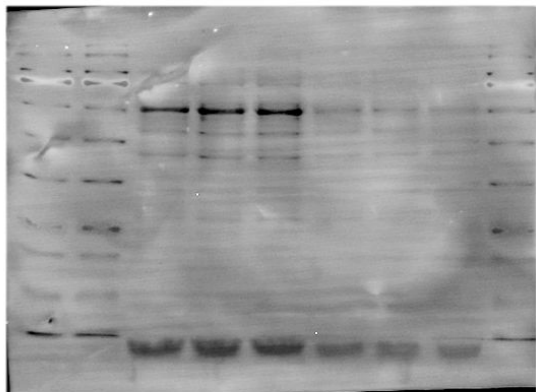

anti MMP-9

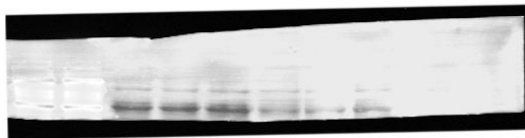

anti MPO

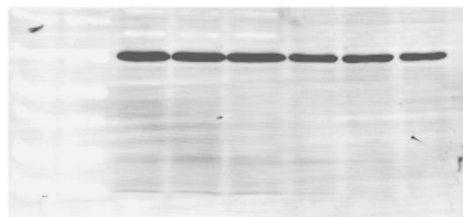

anti NE

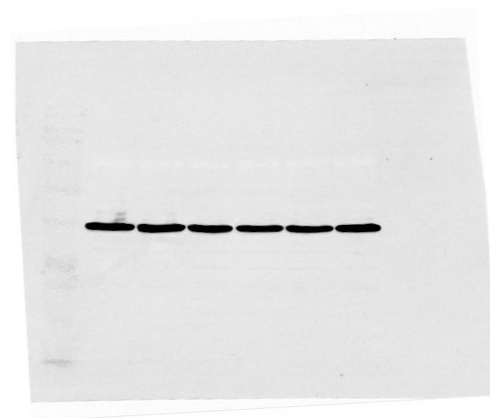

anti GAPDH

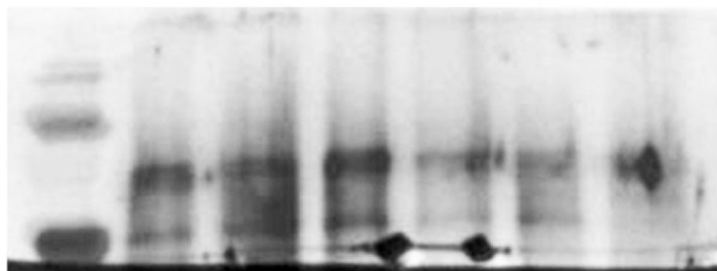

anti PAD-4

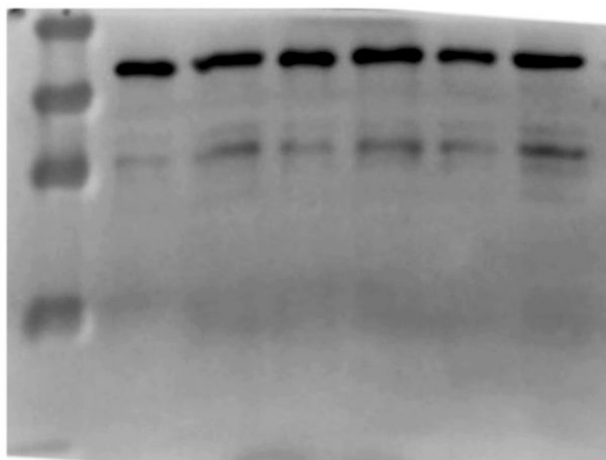

anti GAPDH

Supplement: Supplementary file 2 — Additional file 2. [file 12916_2022_2564_MOESM2_ESM.pdf]
